# Preconcentration and Detection of Gefitinib Anti-Cancer Drug Traces from Water and Human Plasma Samples by Means of Magnetic Nanoparticles

**DOI:** 10.3390/nano10061196

**Published:** 2020-06-19

**Authors:** Hadeer Borg, Dániel Zámbó, Heba Elmansi, Heba M. Hashem, Jenny Jehan Nasr, Mohammed I. Walash, Nadja C. Bigall, Fathalla Belal

**Affiliations:** 1 Department of Pharmaceutical Analytical Chemistry, Faculty of Pharmacy, University of Mansoura, Mansoura 35516, Egypt; Hadeerborg@gmail.com (H.B.); dr_heba85@hotmail.com (H.E.); hebamaher13@yahoo.com (H.M.H.); nasrjj@yahoo.com (J.J.N.); mwalash@yahoo.com (M.I.W.); ffbelal@yahoo.com (F.B.); 2 Department of Pharmaceutical Chemistry, Faculty of Pharmacy, Delta University, Gamasa 35712, Egypt; 3 Institute of Physical Chemistry and Electrochemistry, Leibniz Universität Hannover, 30167 Hannover, Germany; nadja.bigall@pci.uni-hannover.de; 4 Chemistry Department, Faculty of Science, Ain Shams University, Abbasia, Cairo 11566, Egypt

**Keywords:** gefitinib, magnetic solid-phase extraction, iron oxide nanoparticles, human plasma, liquid chromatography

## Abstract

Along of widespread application of anti-cancer drug Gefitinib (GEF), it appears in human body fluids as well as clinical wastewater. Consequently, a reliable and easy-to-adapt detection technique is of essential importance to quantify the drug in different media. The extraction and quantitative detection of anti-cancer drug Gefinitib (GEF) is demonstrated based on a straightforward and efficient magnetic nanoparticle-assisted preconcentration route from water and human plasma samples. Iron oxide magnetic nanoparticles (Fe_3_O_4_) have been prepared with an average particle size of 15 nm and utilized as extractible adsorbents for the magnetic solid-phase extraction (MSPE) of GEF in aqueous media. The method is based on MSPE and preconcentration of GEF followed by High-Performance Liquid Chromatography-Ultraviolet Detection (HPLC-UV). The yield of GEF extraction under the optimum MSPE conditions were 94% and 87% for water and plasma samples, respectively. The chromatographic separation was carried out isocratically at 25 °C on a Phenomenex C8 reversed phase column (150 mm × 4.6 mm, with 5 µm particle size). The proposed method was linear over concentration ranges of 15.0–300.0 and 80.0–600.0 ng/mL for water and plasma samples with limits of detection of 4.6 and 25.0 ng/mL in a respective order. Relative standard deviations (%RSD) for intra-day and inter-day were 0.75 and 0.94 for water samples and 1.26 and 1.70 for plasma samples, respectively. Using the magnetic nanoparticles (MNPs) as loaded drug-extractors made the detection of the anti-cancer drug environmentally friendly and simple and has great potential to be used for different drug-containing systems.

## 1. Introduction

**Figure 1:**
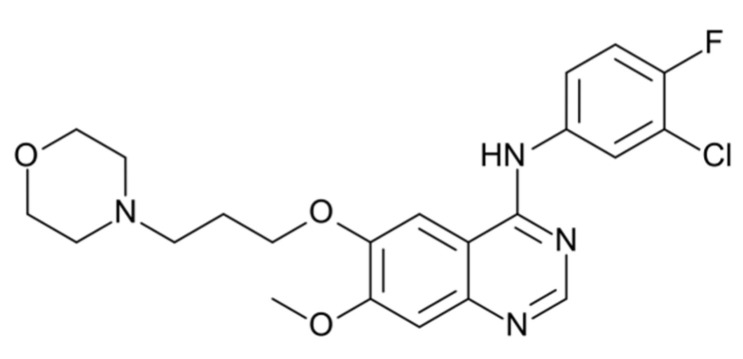
Chemical structure of Gefitinib.

Gefitinib is an anti-tumor drug that is used widespread and plays an important role in cancer therapy. It inhibits the intracellular phosphorylation of numerous tyrosine kinases associated with transmembrane cell surface receptors, including the tyrosine kinases associated with the epidermal growth factor receptor (EGFR-TK). Gefitinib has antineoplastic activity, and has been approved for the treatment of non-small cell lung cancer (NSCLC) [1]. The chemical name of gefitinib is N-(3-Chloro-4-fluorophenyl)-7-methoxy-6-[3-(morpholin-4-yl)propoxy]quinazolin-4-amine corresponding to the molecular formula C_22_H_24_ClFN_4_O_3_. The chemical structure of gefitinib is shown in Figure 1. Gefitinib is described in the Ph. Eur. monograph 2866 [2]. Its solubility in water increases with decreasing pH [2]. The molecule has two pKa of 5.4 and 7.2 and its partition coefficient logP is 3.75 (octanol/water) [2]. IRESSA^®^ (gefitinib tablets) contains 250 mg of gefitinib and is available as brown film-coated tablets for daily oral administration. It is absorbed slowly after oral administration with mean bioavailability of 60%. Elimination is by metabolism (primarily CYP 3A4) and excretion in feces [3]. During the application of Gefitinib, it appears not only in the human body but in clinical wastewater as well; thus, efforts towards the detection and separation of this compound are of great importance.

Consequently, there is an essential demand for the development of sensitive analytical methods for the detection and evaluation of Gefitinib as an important antineoplastic drug with strong potency. Numerous analytical methods have already been developed for the quantitative determination of Gefitinib using spectrophotometry [4,5], High-Performance Liquid Chromatography-Ultraviolet Detection (HPLC-UV) [6,7,8], HPLC-MS/MS [9,10,11] and UHPLC-MS/MS [12]. However, our method is aimed at determining Gefitinib by HPLC-UV after magnetic solid-phase extraction (MSPE), which is a relatively new promising technique for the preparation of analytical samples and has gained considerable attention [13,14,15]. This method opens up new routes towards the separation of drugs from medical wastewater as well as the detection of the anticancer drugs in human samples.

Using MSPE, the preconcentration of analytes from large volumes can be carried out by means of magnetic sorbents. Magnetite nanoparticles (Fe_3_O_4_) are excellent candidates for the above-mentioned purposes and enable a greatly simplified sample pretreatment using MSPE since the phase separation can be quickly and easily accomplished by applying an external magnetic field [13]. In this study, the method for the preparation and the application of aqueous, surfactant-coated Fe_3_O_4_ MNPs as novel sorbents for the SPE of Gefitinib from water and plasma samples. To the best of our knowledge, this is the first study that uses Fe_3_O_4_ NPs as an extraction agent for Gefitinib from water and human plasma samples. Table 1 highlights the main features of our method compared to various, previously reported works.

## 2. Materials and Methods

### 2.1. Chemicals and Reagents

Gefitinib was kindly obtained from Pfizer Inc. (New York, NY, USA) (certified to have a purity of >99%). Ethanol (EtOH, ≥99.8% for HPLC), acetonitrile (ACN, ≥99.9% for HPLC), methanol (MeOH, ≥99.9% for HPLC), iron(II) chloride tetrahydrate (FeCl_2_∙4H_2_O, ≥99.0%), potassium hydroxide (KOH, >85%) and trichloroacetic acid (Cl_3_CCOOH, ≥99.0%) were purchased from Sigma-Aldrich (Darmstadt, Germany). Iron(III) chloride hexahydrate (FeCl_3_∙6H_2_O, >99.0%) was supplied by Fluka (Buchs, Switzerland). Sodium dihydrogen phosphate (NaH_2_PO_4_, ≥98%), sodium dodecyl sulphate (SDS, ≥99%) and orthophosphoric acid (H_3_PO_4_, ≥98%) were obtained from Adwic Chemical Company (Cairo, Egypt). All chemicals and reagents were of analytical grade and used without further modification. Ultrapure water (Milli-Q with a resistivity of 18.2 MΩ∙cm) was used for the synthesis and purification of nanoparticles and double-distilled water was used during the study. Human plasma samples were collected from one patient treated with Iressa tablets at Mansoura University Oncology Centre. Pooled blank plasma was obtained from Mansoura University Hospitals (Mansoura, Egypt) and was kept frozen until use after gentle thawing. The study was conducted in accordance with the Declaration of Helsinki, and the protocol was approved by the Research Ethical Committee (REC) of Faculty of Pharmacy, Mansoura University (Date of REC protocol approval: 27/06/2018) under the code number (2018-47). The research work conducted on human has been approved on 29/03/2020 under the code number: 2020-34. Sample donors signed a consent form approved by the Ethics Committee of Faculty of Pharmacy, Mansoura University.

### 2.2. Measurement Methods

The characterization of the nanoparticles was performed using Zetasizer Nano ZSP (Malvern Panalytical, Malvern, UK) equipped with a 633-nm laser, a JEOL JSM-6700F Field-Emission Scanning Electron Microscope (FE-SEM, operated at 5 kV, Tokyo, Japan) and a Tecnai G2 F20 TMP Transmission Electron Microscope (TEM, operated at 200 kV, FEI, Hillsboro, OR, USA). The crystal structure of the nanoparticles was performed by a Bruker AXS D8 Advance X-ray diffractometer (XRD) using a Cu-Kα_1,2_ radiation source. Chromatographic separations were carried out on a Shimadzu HPLC system (Kyoto, Japan) consisting of a degasser unit (DGU-20 A3R), a pump (LC-20 AT), an injection valve with a 20-µL sample loop, a column oven (CTO-20 A) and a UV detector (SPD-20 A). The separations were carried out on a Hyper Clone C8 column (150 mm × 4.6 mm, with 5 µm particle size) from Phenomenex (Torrance, CA, USA). The mobile phase consisted of acetonitrile: 0.3% phosphoric acid in a ratio of 35:65, the pH was adjusted to 3 with triethylamine. The analysis was done under isocratic conditions at a flow rate of 0.8 mL/min and the detection was performed at 345 nm. For data processing and acquisition, LabSolutions software version 5.84 from Shimadzu was used. A Jenway 6850 UV/Vis Spectrophotometer (Stone, UK) was applied for the absorbance measurements of the solutions. An ultrasonic cleaner set (WUC-D06H) (Seoul, Korea) and a pH Meter from Jenway 3505 (Stone, UK) were used.

### 2.3. Synthesis of Fe_3_O_4_ Nanoparticles

Fe_3_O_4_ nanoparticles were synthesized via the chemical co-precipitation method partly published elsewhere [20] but applying modifications and optimization. Briefly, in a 25-mL volumetric flask, 0.165 g FeCl_2_∙4H_2_O and 0.225 g FeCl_3_∙6H_2_O were dissolved in ultrapure water and completed to the mark. Simultaneously, 25 mL of 0.9 M KOH was prepared. The Fe^2+^/Fe^3+^ mixture was then heated to 80 °C in a rounded three neck flask under N_2_ gas bubbling and vigorously stirred for 15 min. The KOH solution was then added drop wisely (6.25 mL/min) form a black precipitate of Fe_3_O_4_ nanoparticles. The formed precipitate was kept for another 15 min under N_2_ gas at the same temperature. Finally, the obtained Fe_3_O_4_ nanoparticles were collected and washed with 50 mL Milli-Q water four times to remove any residue of KOH and unreacted precursor. The synthesized NPs were characterized by Dynamic Light Scattering (DLS), XRD, FE-SEM and TEM.

### 2.4. Optimum Conditions for MSPE of Gefitinib

The magnetic solid-phase extraction of gefitinib was performed in 5-mL volume Wassermann tubes, 300 µL from Gefitinib stock solution (200.0 µg/mL) was added to 150 µL of sodium dodecyl sulphate (SDS) (2 mg/mL) and then 0.5 mg of Fe_3_O_4_ nanoparticles was added. Finally, the volume was completed to 3 mL with phosphate buffer (pH = 3). The prepared mixture was vortexed for 1 min and then kept for 25 min under sonication for complete extraction. To separate Fe_3_O_4_ nanoparticles from the solution, an external magnet (Nd-Fe-B, (6 × 2 × 1 cm^3^)) was placed under the vial to separate the magnetite while the upper solution was decanted. Finally, Gefitinib was desorbed again from the nanoparticles’ surface by adding 3 mL and 0.5 mL ethanol for UV spectroscopy and HPLC measurements, respectively, followed by sonication for 30 s.

### 2.5. Extraction of GEF from Water and Plasma Samples

The developed MSPE method was applied for the extraction and determination of GEF from water as well as plasma samples. The extraction of GEF from all samples was done according to MSPE procedure described in the “Optimization of MSPE mechanism and procedure” section. However, an additional purification step was carried out on the plasma samples before extraction. A total of 1 mL of [ethanol: 10% trichloroacetic acid (1:1 v/v)] was added to 0.5 mL plasma followed by the centrifugation of the mixture in order to precipitate plasma proteins. MSPE was then applied on the clear supernatant containing the drug. The final elution of the analyte was carried out with 0.5 mL ethanol prior to the injection into the HPLC column. 

## 3. Results and Discussion

### 3.1. Characterization of the Synthesized Fe_3_O_4_ Magnetic Nanoparticles

**Figure 2:**
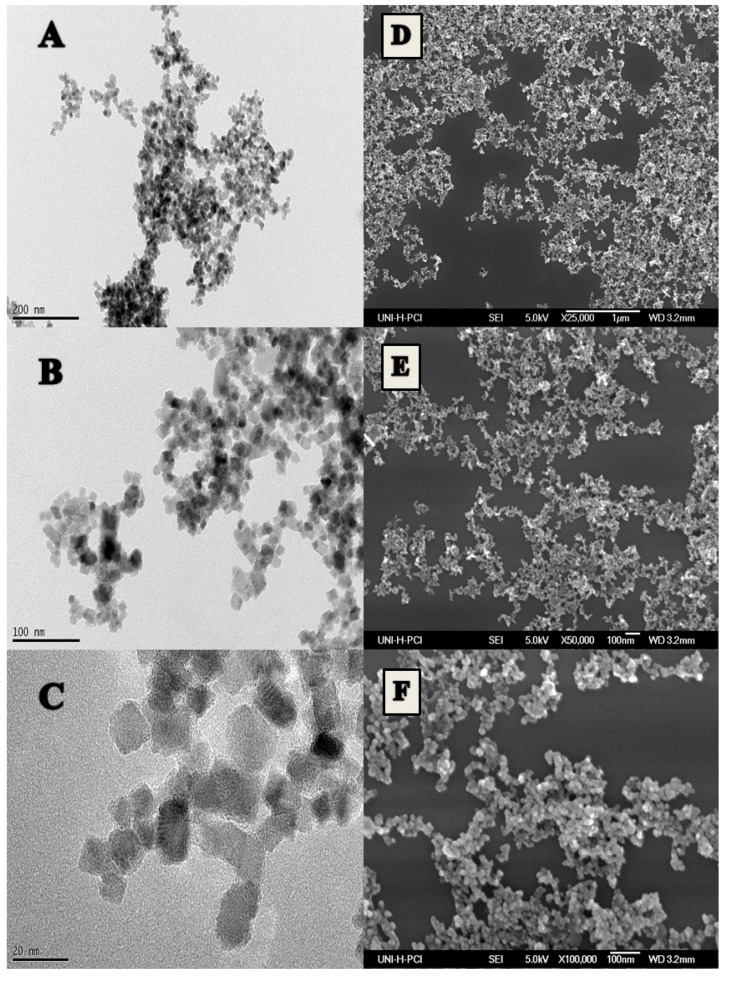
Images of the synthesized iron oxide nanoparticles at different magnifications by (**A**–**C**) a transmission electron microscope (TEM) and (**D**–**F**) a scanning electron microscope (SEM).

**Figure 3:**
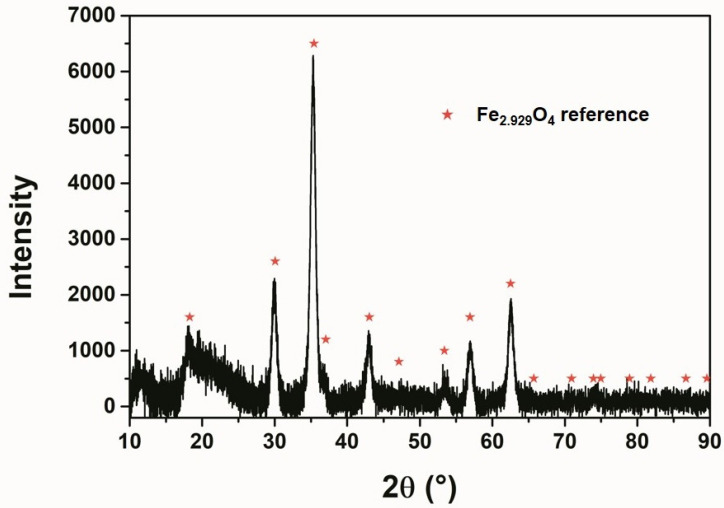
X-ray diffraction (XRD) pattern of the prepared iron oxide MNPs.

The size of the synthesized Fe_3_O_4_ magnetic nanoparticles was characterized by scanning electron microscopy (SEM) and transmission electron microscopy (TEM). In Figure 2, SEM and TEM images show the average particle size of 19 ± 4 nm with narrow size distribution, which was confirmed by DLS as well (Polydispersity index (PdI) = 0.15). This size of the nanocrystals were found to be reproducible over different batches. Figure 3 shows the diffraction pattern of the nanoparticles indicating that the structure of the synthesized nanoparticles matches the reference of magnetite phase (Fe_3_O_4_). The stability of the colloidal solution was proven by measuring the ζ-potential that was found to be +14 mV.

### 3.2. Optimization of MSPE Mechanism and Procedure

**Scheme 1:**
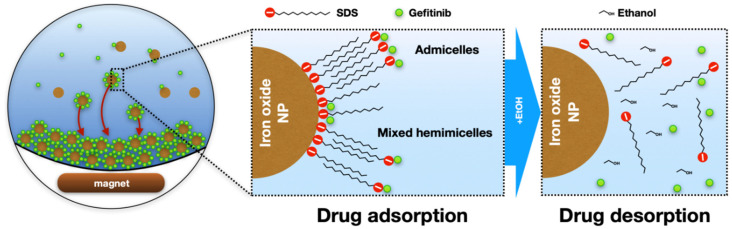
Schematics of the interaction between the analyte and the MNP surface during adsorption and desorption.

The MSPE mechanism was chosen as reported in the previous methods [21,22,23]. It is based on the formation of SDS-coated magnetic nanoparticles’ mixed hemimicelles/admicelles, which was found to be an effective technique for the adsorption of ionic and non-ionic species. There are two possible phenomena for the attraction between the target analyte and the SDS-coated MNPs. The first one relies on the fact that at certain pH, the SDS-coated MNPs possess a negative charge that can electrostatically attract the positively charged analyte at this pH [21]. The second one is due to hydrophobic attraction into the formed hemimicelles/admicelles system [24]. Considering that the zeta potential of the nanoparticles changed from +14 to –20 mV, both mechanisms can be anticipated to take place simultaneously. After loading the MNPs with the target analyte, it can be easily separated from the solution by either centrifugation or applying an external magnet. Finally, the desorption of the analyte from the MNPs’ surface back to the solution can be instantly obtained by adding some organic solvents, such as methanol, ethanol or acetonitrile. The mechanism of the analyte adsorption and desorption can be seen in Scheme 1.

**Scheme 2:**
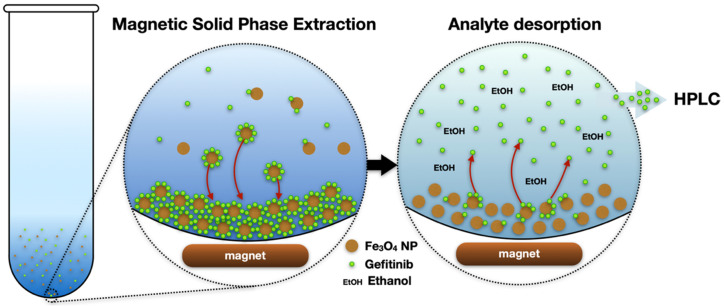
Schematics of the extraction and analysis of Gefitinib using iron-oxide nanoparticles.

Different conditions affecting the MSPE procedure were studied, such as the effect of sample pH, adsorption time, the amount of adsorbent, the amount of SDS, desorption time and the type of desorbing solvent. The effect of the above-mentioned factors was studied based on measuring the absorbance of the sample before and after the adsorption on the SDS-coated MNPs. HPLC-UV was used at the final application step for the validation of the method and for the analysis of the real samples. Series of (5 mL) glass tubes were used throughout the study for easy collection and handling of the samples. Scheme 2 represents the process of the extraction and detection of the analyte.

#### 3.2.1. Effect of the Sample’s pH

**Figure 4:**
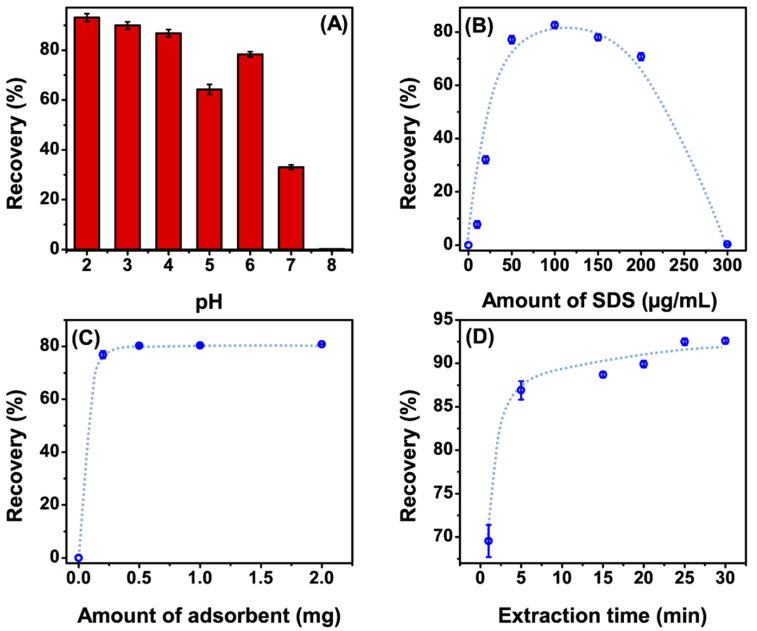
Effect of (**A**) pH; (**B**) the amount of sodium dodecyl sulphate (SDS); (**C**) the amount of adsorbent; and (**D**) extraction time on the extraction efficiency. Blue dashed lines are guides to the eye.

Since the attractive interaction between our target analyte and the SDS-coated MNPs is anticipated to be mainly electrostatic, the pH of the sample plays a critical role in the extraction process. At different pH values, the ionization of Gefitinib and the SDS hemimicelles/admicelles changes [25]. At very low pH, the free protons in the solution can be attracted to the negatively charged hemimicelles/admicelles and decrease its negative charge value, and, as a result, the targeted electrostatic attraction between SDS hemimicelles and the nanoparticles will be declined. Simultaneously, we should consider the ionization of Gefitinib, which was found to possess a full positive charge at pH values less than 6 [25]. On the other hand, at higher pH, OH^−^ ions can be attracted to the surface of Fe_3_O_4_ nanoparticles and hinder the formation of the SDS the hemimicelles/admicelles system [21]. So, theoretically, we have considered the optimum pH for extraction to be in the range of 2 to 4, which equals a Gefitinib charge of +2. We have studied different pH values ranging from 2 to 8 (adjusted by 0.2 M phosphate buffer) as shown in (Figure 4A), and pH 3 was found to be the optimum condition.

#### 3.2.2. Effect of Concentration of SDS

Another critical factor that needed to be carefully studied was the SDS concentration. As was mentioned before, the mechanism of adsorption depends on the formation of SDS hemimicelles or admicelles on the surface of Fe_3_O_4_ nanoparticles, which can be obtained by SDS concentrations above its critical aggregation concentration (CAC) [26]. Different concentrations of the SDS were studied in the range of 10.0–300.0 µg/mL as shown in Figure 4B. In the absence of SDS, GEF hardly adsorbed on the surface of Fe_3_O_4_ nanoparticles. However, the adsorption of GEF on the surface of the SDS-coated MNPs increased with increasing concentrations of SDS to a certain point (point of formation of hemimicelles/admicelles), which was found to be 100 µg/mL. At concentrations greater than 100.0 µg/mL, the adsorbed amount started to decrease gradually until it completely disappeared at a concentration of 300 µg/mL. The reason can be explained as, at higher concentrations, the SDS-micelles started to be formed in the bulk solution, which can compete with the adsorption of GEF. Therefore, 100.0 µg/mL was chosen as the optimum concentration of SDS in later studies. 

#### 3.2.3. Effect of Amount of Adsorbent

The amount of adsorbent added was studied in the range of 0.2–2.0 mg (Figure 4C). The adsorption of GEF gradually increased by increasing the amount of adsorbent until a maximum value at 0.5 mg of MNPs. Above this concentration, increasing the amount of MNPs showed no further increase in the adsorption. Consequently, 0.5 mg was found to be the optimum amount added to extract GEF completely from a 3-mL solution volume.

#### 3.2.4. Effect of Extraction Time

Figure 4D shows the effect of extraction time on the adsorption of GEF in the range of 1–30 min. It can be seen that GEF adsorption increased gradually up to 25 min and then saturated. Based on the characteristic of the adsorption behavior, the optimal extraction time was found to be 25 min.

#### 3.2.5. Desorption Conditions

The desorption of our target analyte from the surface of the SDS-coated MNPs can be obtained by adding organic solvents as reported in previous works [24,27,28,29]. It was found that organic solvents can disrupt the formed hemimicelles/admicelles, which diminishes the available place for GEF adsorption. Acetonitrile, methanol and ethanol was studied as different desorbing solvents, ethanol was chosen as it resulted in the highest extraction efficiency. Desorption time was studied as well; only 30 s was enough for the complete desorption of the analyte.

#### 3.2.6. Extraction Procedure from Spiked Plasma Samples

Under the optimum MSPE conditions, the extraction of GEF directly from spiked plasma samples was challenging. As for a complex matrix such as plasma, there are various components expected to be present (for example proteins, ions, nutrient molecules and wastes) that can compete with the target analyte on adsorption to the surface of the nanoparticles. Therefore, an additional protein precipitation step was added to the procedure. Firstly, solely acetonitrile was used to precipitate plasma proteins; however, the recovery was not satisfactory. On the other hand, using a mixture of ethanol and 10% trichloroacetic acid in a ratio of 1:1 (v/v) resulted in a higher percent recovery reaching 87 ± 1%.

### 3.3. Analytical Performance

The proposed method was applied for the quantitative analysis of the drug in water and plasma samples. Method validation was accomplished by testing linearity and range, the limit of quantification (LOQ), the limit of detection (LOD), accuracy, precision, and specificity according to International Conference on Harmonisation (ICH) recommendations [30]. Table 2 illustrates the quantitative parameters of the proposed method. The calibration curves were linear over the ranges of 15 to 300 and 80 to 600 ng/mL for water and plasma samples, respectively. The LOD values for water and plasma samples were 4.6 and 25 ng/mL, respectively. LOQ and LOD were calculated according to ICH Q2R1 recommendations [30].

LOD = 3.3*Sa*/*b* (1)

LOQ = 10*Sa*/*b* (2)

where *Sa* is the standard deviation of the intercept and *b* is the slope of the calibration curve.

**Figure 5:**
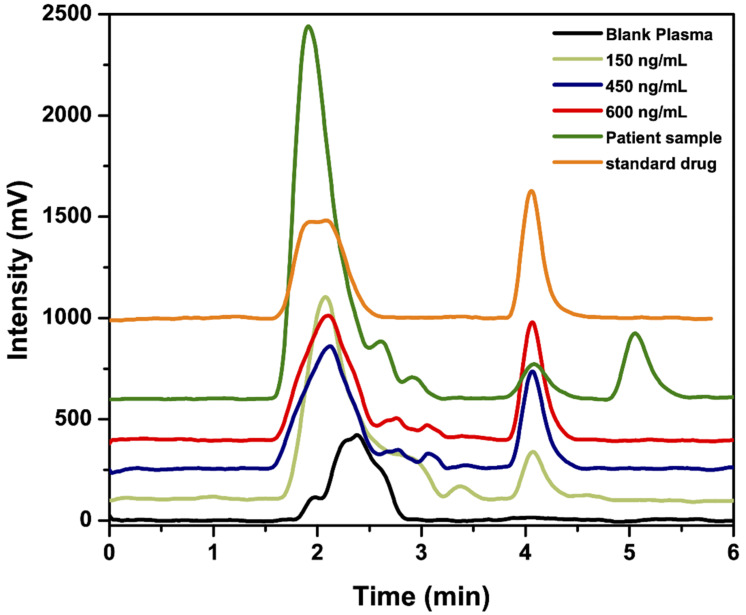
Typical chromatograms for the determination of standard GEF (450 ng/mL), GEF in patient plasma sample and GEF in spiked human plasma samples containing 150, 450 and 600 ng/mL by the proposed method.

Intra-day and inter-day precision were studied using the following three different concentrations: 50.0, 150.0 and 300.0 ng/mL for water samples and 150.0, 450.0 and 600.0 ng/mL for plasma samples. The relative standard deviations (%RSD) for intra-day and inter-day were 0.75 and 0.94 for water samples and 1.26 and 1.70 for plasma samples, respectively (results summarized in Table 3). Method specificity was tested by comparing chromatograms of blank water and plasma samples with the spiked ones, no peaks appeared at the same retention time of GEF. Figure 5 shows typical chromatograms obtained from the determination of GEF by the proposed method in spiked plasma samples. The recovery of the analyte was 94.0 ± 1.0% and 87.1 ± 1.0% from water and plasma samples, respectively. The results indicate high sensitivity, good precision, wide linear range and satisfactory recoveries of the proposed method.

### 3.4. Application of the Proposed Method to the Real Plasma Sample

The proposed method was successfully adapted and applied for the determination of GEF in spiked and real plasma samples with satisfactory recovery equal to 87%. The method was tested for its ability to determine the analyte in a real plasma sample obtained from patient treated with GEF.

The sample was collected at peak plasma concentration (C_max_) [3] after 4 h from administration of a single dose of Iressa tablet (250.0 mg). The developed extraction procedure was applied on the collected plasma sample, and the area under the peak obtained after sample injection into the HPLC column was then used to calculate sample concentration. The unknown sample concentration was 132.0 ng/mL which is found to be within the reported C_max_ range [31] (48.7–324.0 ng/mL).

### 3.5. Comparison with Other Reported Methods

Based on the literature review, the HPLC-UV methodology was mostly used for assessing GEF in bulk and pharmaceutical dosage forms. However, more sensitive methodologies, such as HPLC/MS and UPLC/MS, were frequently used for the determination of our target analyte in complex matrices for example human plasma. The proposed HPLC-UV method showed a wide linear range in both water and plasma samples with very low LODs and LOQs comparable with those obtained from previously reported HPLC/MS methods. Table 1 in the introduction highlights the main features of our method compared to other, previously reported studies.

## 4. Conclusions

A novel SPE method for the preconcentration, detection and quantification of GEF in water and plasma samples based on SDS-coated magnetic nanoparticles mixed hemimicelles/admicelles was developed. The targeted analyte was collected and extracted from water and human plasma samples by means of aqueous Fe_3_O_4_ nanoparticles acting as easily extractable carriers loaded by the drug on their surface. The extraction method is clean and environmentally friendly, as it does not use any toxic, hazardous or expensive materials. Through the magnetic features and high surface-to-volume ratio of MNPs, the separation procedure was simple and resulted in high extraction capacity within short time using a straightforward and simple magnet. Sample preconcentration prior to HPLC-UV analysis as well as the simple and effective drug desorption resulted in greatly reduced LODs and LOQs compared to other reported methods. The proposed method is sensitive, simple and suitable for the fast determination of GEF in water and plasma samples and opens up possibilities to be extended for extracting and detecting different drugs in various media.

## Figures and Tables

**Table 1:** Comparison of the proposed method with other reported methods to determine Gefitinib (GEF) in different sample matrices.

Matrix	Extraction Method	Detection System	Extraction Time (min)	LOD (ng/mL)	Linear Range (ng/mL)	%RSD	Ref.
Water	SPE	HPLC-UV	20	4.6	15–300	1.0	This work
Human Plasma	SPE	HPLC-UV	20	25.0	80–600	1.1	This work
Polymeric nano-formulation	-	HPLC-UV	-	37.8	200–1200	<1	[6]
Tablet	-	HPLC-UV	-	90	5000–25,000	0.877	[16]
Human Plasma	LLE	HPLC-MS	15	-	5–3000	<5	[17]
Human Plasma	Protein precipitation	HPLC-MS	10	-	1–1000	<15	[18]
Rat Plasma	Protein precipitation	HPLC-MS	10	-	5–1000	<15	[18]
Human Plasma	LLE	LC-MS/MS	>30	-	0.5–1000	<15	[19]

LOD: limit of detection; RSD: relative standard deviation; SPE: solid-phase extraction; LLE: liquid–liquid extraction.

**Table 2:** Analytical performance data of the proposed method for extraction and determination of GEF from water and plasma samples.

Matrix	LOD (ng/mL)	LOQ (ng/mL)	%RSD		%Error	Extraction Percentage (%)	Linear Range (ng/mL)	R^2^
			Intra-Day	Inter-Day				
Water	4.6	15.0	0.8	0.9	0.55	94.0	15–300	0.9999
Human plasma	25.5	80.0	1.3	1.7	0.60	87.1	80–600	0.9997

**Table 3:** Precision data for the determination of GEF in water and plasma samples by the proposed method.

		Intra-Day Precision			Inter-Day Precision		
		Conc. Taken (ng/mL)	Conc. Found (ng/mL)	% Found	Conc. Taken (ng/mL)	Conc. Found (ng/mL)	% Found
Water samples		50.00	50.62	101.24	50.00	50.62	101.24
		150.00	151.99	101.33	150.00	152.63	101.75
		300.00	299.97	99.99	300.00	299.76	99.92
	$\bar{X}$ ± SD	100.85 ± 0.75			100.95 ± 0.94		
	%RSD	0.75			0.94		
	%Error	0.43			0.54		
Plasma samples		150.00	147.76	98.51	150.00	149.86	99.91
		450.00	454.54	101.01	450.00	452.43	100.54
		600.00	596.94	99.49	600.00	584.16	97.36
	$\bar{X}$ ± SD	99.67 ± 1.26			99.27 ± 1.70		
	%RSD	1.26			1.70		
	%Error	0.73			0.98		

N.B. Each result is the average of three separate determinations.
